# Off-label drug use in children and adolescents treated with antidepressants and antipsychotics: results from a prospective multicenter trial

**DOI:** 10.1186/s13034-025-00957-7

**Published:** 2025-10-13

**Authors:** Regina Taurines, Manfred Gerlach, Christoph U. Correll, Paul L. Plener, Uwe Malzahn, Peter Heuschmann, Maike Scherf-Clavel, Hans Rock, Wolfgang Briegel, Christian Fleischhaker, Alexander Häge, Tobias Hellenschmidt, Hartmut Imgart, Michael Kaess, Andreas Karwautz, Michael Kölch, Karl Reitzle, Tobias J. Renner, Su-Yin Reuter-Dang, Christian Rexroth, Gerd Schulte-Körne, Frank Theisen, Elvira Tini, Christoph Wewetzer, Stefanie Fekete, Marcel Romanos, Karin M. Egberts

**Affiliations:** 1 Department of Child and Adolescent Psychiatry, Psychosomatics and Psychotherapy, Center of Mental Health, University Hospital of Wuerzburg, Margarete-Hoeppel-Platz 1, 97080 Wuerzburg, Germany; 2 Department of Child and Adolescent Psychiatry, Charité Universitaetsmedizin Berlin, 13353 Berlin, Germany; 3 Department of Psychiatry, The Zucker Hillside Hospital, Northwell Health, Glen Oaks, NY 11004 USA; 4 Department of Psychiatry and Molecular Medicine, Donald and Barbara Zucker School of Medicine at Hofstra/Northwell, Hempstead, NY 11549 USA; 5 German Center for Mental Health (DZPG), Partner Site Berlin, 10117 Berlin, Germany; 6 Department of Child and Adolescent Psychiatry and Psychotherapy, University Hospital of Ulm, 89075 Ulm, Germany; 7 Department of Child and Adolescent Psychiatry, Medical University Vienna, Vienna, Austria; 8 Clinical Trial Center Wuerzburg, University Hospital Wuerzburg, 97080 Wuerzburg, Germany; 9 Institute of Clinical Epidemiology and Biometry, University of Wuerzburg, 97080 Wuerzburg, Germany; 10 Institute of Medical Data Science, University Hospital Wuerzburg, Wuerzburg, Germany; 11 Department of Psychiatry, Psychosomatics and Psychotherapy, Center of Mental Health, University Hospital Wuerzburg, 97080 Wuerzburg, Germany; 12 Central Information Office, Department of Neurology, Philipps University of Marburg, Marburg, Germany; 13 Department of Child and Adolescent Psychiatry, Psychosomatics and Psychotherapy, Leopoldina Hospital, Schweinfurt, Germany; 14 Department of Psychology, Bielefeld University, 33615 Bielefeld, Germany; 15 Department of Child and Adolescent Psychiatry and Psychotherapy, University Medical Center Freiburg, Freiburg, Germany; 16 Department of Child and Adolescent Psychiatry and Psychotherapy, Medical Faculty Mannheim, Central Institute of Mental Health, Heidelberg University, Mannheim, Germany; 17 Department of Child and Adolescent Psychiatry, Psychotherapy and Psychosomatic Medicine, Vivantes Clinic Berlin Neukoelln, Berlin, Germany; 18 Parkland-Clinic, Clinic for Psychosomatics and Psychotherapy, Academic Teaching Hospital for the University Gießen, 34537 Bad Wildungen, Germany; 19 Clinic for Child and Adolescent Psychiatry, Center for Psychosocial Medicine, University Hospital Heidelberg, Heidelberg, Germany; 20 University Hospital of Child and Adolescent Psychiatry and Psychotherapy, University of Bern, Bern, Switzerland; 21 Department of Child and Adolescent Psychiatry and Psychotherapy, Medical School Brandenburg, Neuruppin, Germany; 22 Department of Child and Adolescent Psychiatry, Neurology, Psychosomatics, and Psychotherapy, University Medical Center Rostock, Rostock, Germany; 23 German Center for Child and Adolescent Health (DZKJ), Partner Site Greifswald/Rostock, 18147 Rostock, Germany; 24 Specialist practice and Medical Service Center for Child and Adolescent Psychiatry Munich, Munich, Germany; 25 Department of Child and Adolescent Psychiatry, Psychosomatics and Psychotherapy, University Hospital of Psychiatry and Psychotherapy Tuebingen, Center of Mental Health Tuebingen, 72076 Tuebingen, Germany; 26 German Center for Mental Health (DZPG), Partner Site Tuebingen, 72076 Tuebingen, Germany; 27 Clinic for Child and Adolescent Psychiatry, Psychosomatics and Psychotherapy at the Regensburg District Hospital, Medbo KU, University Hospital, Regensburg, Germany; 28 Department of Child and Adolescent Psychiatry, Psychosomatics and Psychotherapy, Ludwig-Maximilians-University (LMU) Hospital, Munich, Germany; 29 Department of Child and Adolescent Psychiatry and Psychotherapy, Herz-Jesu-Krankenhaus gGmbH, Fulda, Germany; 30 Department of Child and Adolescent Psychiatry and Psychotherapy, University Hospital of Psychiatry Zurich, Zurich, Switzerland; 31 Clinic for Child and Adolescent Psychiatry and Psychotherapy, Clinics of the City Cologne GmbH, 51109 Cologne, Germany

**Keywords:** Off-label drug use, Children, Adolescents, Antidepressants, Antipsychotics, Depression, Obsessive compulsive disorder, Schizophrenia

## Abstract

**Background/Objectives:**

Off-label psychopharmacologic medication use is widespread in child and adolescent psychiatry, but little is known about its associated factors. This study aimed to assess frequency and determinants of off-label use of antidepressants and antipsychotics in youths in daily clinical practice.

**Methods:**

In a prospective clinical study (‘TDM-VIGIL’) at 18 centers in three German-speaking countries, child psychiatric patients aged 4–18 years undergoing routine treatment with antidepressants and antipsychotics were systematically followed. Demographic, clinical and pharmacological data were collected in an online-based patient registry; off-label use was categorized by reasons, including age, indication or duration of treatment for each treatment episode. Examined correlates of off-label use included sex, treatment setting, diagnosis and illness severity.

**Results:**

About 67% of all antidepressant and antipsychotic treatment episodes in the 700 included patients (mean age = 14.6 years, girls = 67%) were off-label. For antidepressants, 55.2% were off-label (age = 51.1%, non-licensed indications = 37.4%, age + indication = 11.5%), for antipsychotics 81.7% were off-label (age = 29.4%, non-licensed indications = 33.2%, age + indication = 37.4%). Sex, age (< 12, ≥ 12 years) as well as illness severity were not associated with off-label use. In antidepressant treatment, ‘depression’ and ‘obsessive compulsive disorder’ diagnoses were associated with reduced and ‘suicidality at admission’ with increased off-label prescriptions. In antipsychotics, ‘schizophrenia diagnoses’ was linked to decreased, university hospital treatment to increased off-label use.

**Conclusions:**

The frequency of off-label use of antidepressants and even more of antipsychotics in youths treated at specialized child psychiatric centers is high. As the clinical efficacy and safety of off-label antidepressant and antipsychotic use in youth is under-researched, our results call for further pharmacovigilance studies and strategies to improve drug safety.

## Background

Treatment with psychotropic drugs is often an essential component of the multimodal therapy of children and adolescents with severe mental illnesses [1]. Recent studies have shown an increase in prescriptions of psychotropic drugs in this age group [2, 3]. However, only a small number of substances are licensed for this age group, and the approval status varies from country to country. “On-label” use thereby is defined by the prescription of an approved medicinal product for which efficacy, safety and appropriate pharmaceutical quality are tested as part of the approval procedure. Authorization is only granted for those areas of application (indications) and age range for which efficacy and safety have been demonstrated in the authorization documents. However, the lack of detailed knowledge from registration studies about age-specific pharmacokinetics, optimal dosing or the benefit-risk-ratio for many psychotropic drugs bears the risk of suboptimal dosing, intoxication, ineffective therapy and adverse drug reactions [4–6]. Since pediatric patients nevertheless cannot be denied psychopharmacotherapy for ethical reasons if there is a therapeutic need, it is common practice that psychotropic drugs are administered ‘off-label’, i.e. outside of the marketing authorization with regard to age, indication or treatment duration, highlighting the need for more research and economic and legal incentives to encourage companies to test and register off-patent drugs for children.

The frequency, extent and consequences of off-label use in children and adolescents (‘youths’) are subject of debate, as data are limited and very heterogeneous due to different study populations, study designs and underlying definitions of off-label use [7]. Additionally, international literature comparisons are complicated due to differences in health care systems and marketing authorizations even between European countries [8]. Studies from different parts of the world have found a high proportion of off-label use of antidepressants among minors, ranging from 42% [9] to 91% [10–13], with a decline in frequency over time [9, 10, 14–23].

The most recent German retrospective study on off-label use of antidepressants in an unselected sample of minors using data from statutory health insurance funds found decreasing percentages (from 58.0 to 40.9%) of antidepressant off-label prescriptions in the outpatient setting from 2004 to 2011 [24, 25]. The observed decrease in off-label use could be understood in the context of the expanded approval of fluoxetine in 2006 for the treatment of moderate and severe depressive episodes in children older than 8 years of age and a subsequent increase in on-label prescriptions of fluoxetine for pediatric depression. In a recent study in a child psychiatric hospital in Germany, off-label use in adolescents hospitalized due to acute suicidality was 31% for antidepressants [26].

For antipsychotics, high rates of off-label prescriptions in minors have been documented, too [27, 28], as they are used for a variety of indications other than psychosis or mania or irritability associated with autism, including tic disorders, sleep disturbances, unipolar or bipolar depression, impulsive-aggressive behavior, or hyperactivity and obsessional thinking in patients with anorexia nervosa [29]. In contrast to antidepressants, in many western countries a stable or even increasing antipsychotic prescription rate with a high percentage of off-label use has been reported [21, 30–48], especially in some specific countries, e.g. in Iceland [9] or specific populations, e.g. patients with anorexia nervosa for whom no psychotropic drugs are licensed [27].

The majority of these published data on off-label prescriptions rests on information from insurance data, self-reported surveys, or community and localized pharmacy-dispensing data. These data sources are often restricted to specific social or regional groups, which may impair solid conclusions [31, 49–59]. Apart from a recent Swiss study [60] only very few systematic clinical studies on the off-label use of psychotropic drugs in minors have been conducted to date. To our knowledge, the present study is the first multicenter study in child and adolescent psychiatry to investigate the (off-label) use of antidepressants and antipsychotics and type of off-label use in children and adolescents treated in daily clinical practice within a prospective clinical trial (‘TDM-VIGIL’). As recruitment took place in specialized child psychiatric settings, accuracy of diagnostics and treatment strategy could be expected to be much higher compared to former studies on insurance drug dispensation data. TDM-VIGIL data were collected using a secure online patient registry allowing the inclusion of more detailed individual clinical patient data. The aim of this study was to characterize the frequency and types of off-label prescribing of antidepressants and antipsychotics in youth as well as to identify potential correlates, such as age, sex, diagnosis, severity of disorder or treatment setting (outpatient versus inpatient; university versus non-university center).

## Subjects and methods

### Setting and study population

The present study on the off-label use of antidepressants and antipsychotics of youth treated in daily clinical practice was part of the ‘TDM-VIGIL study’ (EudraCT 2013-004881-33) funded by the German Federal Institute for Drugs and Medical Devices (BfArM-code: V-15322/68605/2013–2018). The study design as well as primary and other secondary outcome measures have been described before [6].

In brief, within this multicenter prospective follow-up study, data were collected via an internet-based patient registry from October 2014 to December 2018 at 10 university hospitals, 7 child and adolescent psychiatric state hospitals in Germany, Switzerland and Austria, and one private specialist practice, forming the TDM-VIGIL consortium [6, 61]. All study centers were members of the competence network for TDM in child and adolescent psychiatry (for details see [62]).

All inpatients, day-unit patients and outpatients aged 4–18 years, for whom the treating child and adolescent psychiatrist intended to start a new treatment with an (or another, i.e. switchers) antidepressant or antipsychotic medication, were screened for possible study participation. Exclusion criteria were an absolute clinical contraindication for the chosen antidepressant and antipsychotic and participation in another clinical trial. After providing oral and written information of the patient and the legal guardians, written informed consent was obtained. In the present study, all patients on antidepressants and/or antipsychotics (*n* = 700) of the total of all participants in TDM-VIGIL (*n* = 710) were included.

Patients’ diagnoses were coded clinically by a consultant child and adolescent psychiatrist according to the International Classification of Diseases, 10th Revision (ICD-10-GM). The Clinical Global Impression Scale Severity (CGI-S) [63], was used to assess illness severity. Patients were followed at a minimum of five time points, from baseline before starting treatment with an antidepressant/antipsychotic to a follow-up six months after discharge or end of outpatient treatment (inpatients/day hospital patients were followed as outpatients after discharge).

### Study medication and off-label use

Any clinically used antidepressant or antipsychotic was eligible as the studied psychotropic medications. The study protocol had no influence on the selection of the active substance, formulation, dosing regimen and frequency, nor the duration of use, as this was an observational study. Medication was prescribed upon clinicians’ choice either on- or off-label, as mono- or polypharmacy, based on clinical judgement. Pharmacological agents were defined according to their Anatomical Therapeutic Chemical (ATC)-code (World Health Organization): antidepressants (ATC group N06A), antipsychotics (ATC group N05A). Each treatment episode of the chosen antidepressants and antipsychotics was retrospectively classified by special study investigators (K.E., R.T.) as either on-label or off-label according to the approval status published in the summary of product characteristics (SPCs) in Germany (Table 1). A ‘treatment episode’ refers to the documented period of administering the same active substance (e.g., the interval between the start of the medication and its cessation), either for the first time or after reintroduction, in the same or in different doses, and with a maximum of one day interruption. A ‘treatment episode’ includes baseline documentation without the specific active substance and with the recording of clinical effects after titration. Monotherapy or combination therapy may be present during a ‘treatment episode’.

We defined four categories of off-label use: (1) An episode was assessed as ‘off-label by age’ if the age of the patient was not in accordance with the age-range the drug was licensed for; the lowest age limit was used if SPCs of generic preparations gave inconsistent information or if the approved age varied by indication; (2) ‘off‐label by indication’: if symptoms or illnesses from the patients differed from the approved ones in Germany; (3) ‘off-label by treatment duration’: In risperidone users, if treatment duration was longer than the licensed short-term therapy for disruptive behavior symptoms of 6 weeks; (4) ‘off-label by age and indication’, if criteria (1) and (2) were met at the same time. The off-label use could therefore be due to more than one category. Polypharmacy, defined as the simultaneous administration of at least two psychotropic drugs, was not considered as off-label use per se.

For the off-label use calculations, unless explicitly stated otherwise, treatment episodes were used as the units of analysis and substances were grouped into antidepressants with (1) selective serotonin reuptake inhibitors (SSRIs) and selective noradrenaline-serotonin reuptake inhibitors (SNRIs), (2) tricyclic antidepressants (TCAs), and (3) other antidepressants, and into antipsychotics with (1) second-generation antipsychotics (SGAs), and (2) first-generation antipsychotics.

The following patient- and setting-specific variables were included as possible correlates of off-label use: sex, age (children < 12 years and adolescents ≥ 12 years at inclusion), intelligence level (subaverage yes/no), psychiatric diagnoses, suicidality at baseline (yes/no), treatment setting (university versus non-university; in/out/day patient setting), and the chronological number of the medication episode, i.e., which documented medication episode of the patient it concerns (first, second, 3rd etc.).

**Table 1:** Approval status of antidepressants and antipsychotics in Germany, prescribed in TDM-VIGIL (at time of study)

Psychopharmacologic agent	Age	Indication
ANTIDEPRESSANTS		
Agomelatine	-	-
Amitriptyline	6	Nocturnal enuresis
Citalopram	-	-
Clomipramine	5	Enuresis in context of an overall therapeutic concept
Doxepine	12+	Depressive disorders, anxiety, withdrawal syndromes; restlessness, anxiety or sleep disorders in the context of depression or withdrawal
Duloxetine	-	-
Escitalopram	-	-
Fluoxetine	8+	Moderate to severe episodes of a major depression
Fluvoxamine	8+	Obsessive-compulsive disorder
Mirtazapine	-	-
Paroxetine	-	-
Sertraline	6+	Obsessive-compulsive disorder
Trazodone	-	-
Trimipramine	-	-
Venlafaxine	-	-
ANTIPSYCHOTICS		
Amisulpride	-	-
Aripiprazole	13+ 15+	Manic episodes of bipolar disorder I, maximum 12 weeks Schizophrenia
Chlorprothixen	3+	Psychomotor agitation, manic symptoms
Clozapine	16+	Treatment-resistant schizophrenia
Haloperidol	6+ 10+ 13+	Treatment-resistant significant and persistent aggression in subjects with autism-spectrum disorders Tic disorder Treatment-resistant schizophrenia
Levomepromazine	16	Psychomotor agitation and excitement states in the context of psychotic disorders and manic episodes
Melperone	12+	Sleep disorders, states of confusion, psychomotor agitation and excitement states
Olanzapine	-	-
Paliperidone	15+	Schizophrenia
Pipamperon	‘children’	Sleep disorder, psychomotor excitement states, use in children under close use-risk-balancing
Promethazine	3+	Agitation und excitement states in the context of a geriatric diseases, nausea and vomiting, sleeping disorders
Prothipendyl	-	-
Quetiapine	-	-
Risperidone	5+	Symptomatic short-term treatment (up to 6 weeks) of persistent aggression in conduct disorder in children with subaverage intellectual functioning or mental retardation diagnosed according to DSM-IV criteria; as part of a more comprehensive treatment program
Tiaprid	-	-
Zuclopenthixol	-	-

### Data collection and statistical analysis

For details on the internet-based patient registry (secuTrial^®^-system) and data collection in the electronical Case Report Forms (eCRFs) see [6]. Data monitoring was provided by the Center for Clinical Studies at the University Hospital Wuerzburg. The sample distributions of patients’ characteristics, variables and scores, which were recorded as quantitative data on ordinal, nominal or ratio scales, were described by appropriate summary statistics as location parameters and measures of variation. Summary statistics were given stratified by age cohorts, sex, medication groups, and visits. Interval estimates for proportions of off-label use were given as 95% confidence intervals (95%CIs) using the Wilson score method. We compared distributions, proportions and mean values between independent groups (Type III F-test, tests of the Wald-type within parametric model based analysis) or between paired samples (Friedman-test, Wilcoxon-signed rank test). Categorical data were displayed in contingency tables and, where applicable, chi-square statistics were used to test whether there was an association between variables or groups. Statistical significance was defined as *p* < 0.05 (two-sided) without adjustments for multiple testing. For binary endpoints, especially the indicator for off-label use of drugs, two kinds of modellings were used. For analyses at patient level, e.g. with endpoint ‘at least one episode with off-label drug use in the study’, single-level models of backward stepwise multivariable binary logistic regression analyses were used. For this purpose, the most frequent psychiatric ICD10-diagnoses were grouped into six categories: ‘depressive disorders’, ‘schizophrenia-spectrum disorders’, ‘anxiety disorders’, ‘eating disorders’, ‘obsessive-compulsive disorder (OCD)’, and ‘hyperkinetic disorders’. For analyses at the level of individual medication episodes with endpoint ‘off-label antidepressant use for this episode’, and ‘off-label antipsychotic use for this episode‘, single-level models of backward stepwise multivariable binary logistic regression analyses were used.

Within a medication episodes-based modelling, the question of a possible association between severity of illness (assessed by the CGI-S scale) and subsequent off-label use was investigated by using hierarchical binary logistic regression analyses. In order to achieve sufficient cell counts of on- and off-label use per factor level, the CGI-S assessment variable was recoded resulting in 3 instead of 7 factor levels: (1) normal (not ill) to moderately ill (CGI-S = 1–4), (2) markedly ill (CGI-S = 5), and (3) severely to most extremely ill (CGI-S = 6–7). Medication episodes were matched with the next preceding CGI-S assessment, with a maximum of 10 days between the CGI-S assessment and the start of the medication episode.

Statistical analysis was carried out using the software IBM SPSS-Statistics 25, SAS 9.4, and R v4.0.4 (https://www.R-project.org).

## Results

### Characteristics of the study population

Altogether, 700 patients (66.6% girls, 77.4% inpatients, 8.6% children < 12 years) with a mean age of 14.6 (SD 2.2, range 6–18) were included and followed for a mean of 5.1 months (range 1-936, median = 168.8, interquartile range = 55–241 days). For these patients, a total of 1.265 treatment episodes with an antidepressant and/or antipsychotic were documented with an average of 1.8 episodes (range 1–14, median 1.0). Patient characteristics are summarized in Table 2.

**Table 2:** Characteristics of the study population (*n* = 700)

Sex *n* (%)	
Female	466 (66.6)
Male	234 (33.4)
Age (years), mean (SD), range	14.6 (2.2), 6–18 years
Female, mean (SD)	14.9 (1.7)
Male, means (SD)	14.1 (2.9)
Subjects < 12 years, n (%)	60 (8.6)
Subjects ≥ 12 years, n (%)	640 (91.4)
Setting n (%)	
Inpatient	542 (77.4)
Day-clinic patient	103 (14.7)
Outpatient	55 (7.9)
Anthropometric data, range	
Height	1.14–1.93 m
Weight	19.9–135.0 kg
BMI	10.8–43.3 kg/m^2^
Intelligence level	
Average	633 (90.4)
Sub-average	58 (8.3)
Unknown	9 (1.3)
Suicidality at admission	
Yes	177 (25.3)
No	507 (72.4)
Unknown	16 (2.3)
Most common ICD diagnoses at baseline, n (%) of multiple entries	
F32.1 moderate depressive episode	277 (39.6)
F50.0 anorexia nervosa	111 (15.9)
F32.2 severe depressive episode	90 (12.9)
F42.2 obsessive-compulsive disorder	52 (7.4)
F40.1 social phobia	51 (7.3)
F90.0 attention-deficit/hyperactivity disorder	40 (5.7)
F90.1 hyperkinetic conduct disorder	37 (5.3)
F33.1 recurrent depressive disorder	35 (5.0)
F43.1 post-traumatic stress disorder	27 (3.9)
F20.0 paranoid schizophrenia	24 (3.4)
F50.2 bulimia nervosa	22 (3.1)
Severity of illness (CGI-S), n (%)	
Not assessable	19 (2.7)
Not at all ill	0 (0.0)
Borderline mentally ill	0 (0.0)
Mildly ill	6 (0.9)
Moderately ill	77 (11.0)
Markedly ill	330 (47.1)
Severely ill	232 (33.1)
Extremely ill	29 (4.1)
Missing	7 (1.0)
Polypharmacy, n (%)	305 (43.6)
Off-label use, patients, n (%)	490 out of 700 (70%)
Female, mean (SD)	325 out of 466 (69.7)
Male, means (SD)	165 out of 234 (70.5)
Subjects < 12 years, n (%)	40 out of 60 (66.7)
Subjects ≥ 12 years, n (%)	450 out of 640 (70.3)
Duration of study monitoring, days, median (range)	168.8 (1–936)

CGI-S = Clinical Global Impression Scale - severity, n = number, SD = standard deviation

### Naturalistically prescribed antidepressants and antipsychotics

In total, 15 different antidepressants and 16 antipsychotics were prescribed. Among the various antidepressants, SSRI and noradrenergic and specific serotoninergic antidepressants (79.9%) were most often used – with fluoxetine (49.9% of all antidepressants), sertraline (20.4%) and mirtazapine (14.0%) being the most common ones. SGAs were the predominantly prescribed antipsychotics (80.5%), most frequently aripiprazole (27.3%), quetiapine (21.0%), olanzapine (18.8%), and risperidone (9.4%). Polypharmacy was very common, as 43.6% of the patients were treated with more psychotropic medications simultaneously than one antidepressant and/or one antipsychotic. The vast majority of patients (70.0%) received at least one antidepressant or antipsychotic drug in off-label use over the course of the study (70.5% of the boys and 69.7% of girls). Altogether, 40 of 60 (66.7%) subjects aged < 12 years and 450 out of 640 (70.3%) of adolescents had at least one treatment episode classified as off-label.

### Frequency of off-label use (related to number of medication episodes)

**Fig. 1:**
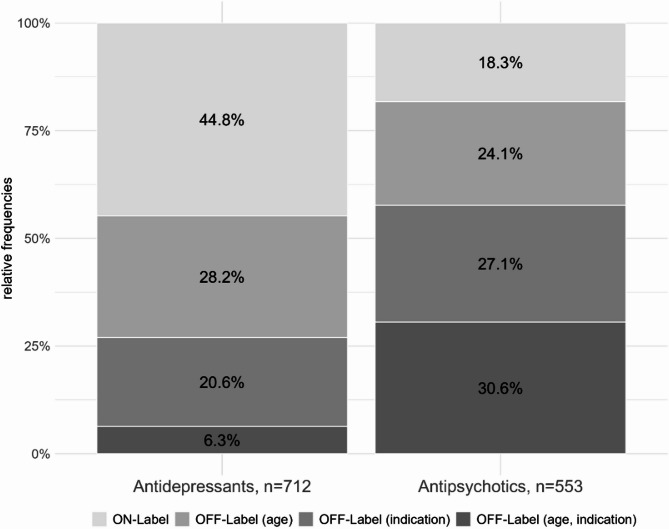
Proportion of off-label prescriptions for antidepressants and antipsychotics

Overall, about two thirds (*n* = 845, 66.8%) of all documented psychotropic drug treatment episodes were classified as off-label; off-label use was observed in 55.2% of all episodes in antidepressants and in 81.7% of antipsychotics (see Fig. 1). The main cause of off-label use in the overall group of medicines studied was off-label use by age (*n* = 334, 39.5%), followed by off-label indication (*n* = 297, 35.1%). Off-label use due to both age and indication was observed in 214 (25.3%) of all antidepressant and antipsychotic episodes.

### Off-label use of antidepressants

In antidepressant drugs, 55.2% (*n* = 393) of all treatment episodes (*n* = 712) were off-label, occurring slightly more frequently in subjects ≥ 12 years (55.5%) than in subjects < 12 years old (50.0%). Altogether, 67.7% of the off-label episodes (versus 82.1% of the on-label episodes) with antidepressants involved patients diagnosed with a depressive episode, which was in general the most common diagnosis in the entire sample. In total, 32.1% of the off-label antidepressant treatment episodes included patients with eating disorders and 30.3% were observed in patients for whom acute suicidality was documented at baseline.

Off-label use based on age alone (*n* = 201, 51.1%) was more common than off-label prescriptions based on indication alone (*n* = 147, 37.4%). All in all, 45 (11.5%) of the treatment episodes with antidepressants were administered outside the approved age and indication.

Of the treatment episodes which were classified as off-label by age (only by age as well as by age and indication), 98.4% were documented in adolescents ≥ 12 years, while only 1.6% were observed in children < 12 years (*n* = 4, age 9, 10, twice 11 years).

### Selective serotonin reuptake inhibitors/Serotonin-norepinephrine reuptake inhibitors/serotonergic substances

The frequency of off-label use in treatment episodes with an SSRI or SNRI (*n* = 591) was 47.2% (*n* = 279), of which 40.1% were in relation to age alone (87.5% of these were episodes in girls), 52.7% in relation to indication (70.1% episodes in girls), and 7.2% in relation to age and indication (60.0% in girls).

The most frequently prescribed antidepressant, fluoxetine, was administered off-label in only 16.1% of the fluoxetine-treatment episodes, and this was exclusively in relation to the indication (70.8% of this were episodes in girls). Sertraline, the second most common antidepressant, was administered off-label in 66.2% of sertraline-treatment episodes (all in relation to indication, 68.8% episodes in girls). Some of the most frequently used substances (escitalopram, citalopram) do not have any approval in children and adolescents and were used off-label at 100%.

### Tricyclic and ‘other’ antidepressants

Treatment episodes (*n* = 17) with a TCAs were off-label in 58.8%, all in relation to age (60.0% of the episodes were documented in girls). All ‘other antidepressants’ (n = 104 episodes) (mirtazapine and agomelatine) were prescribed 100% off-label. Of these, 76.0% were off-label due to age alone, and 24.0% were off-label due to age and indication.

**Fig. 2:**
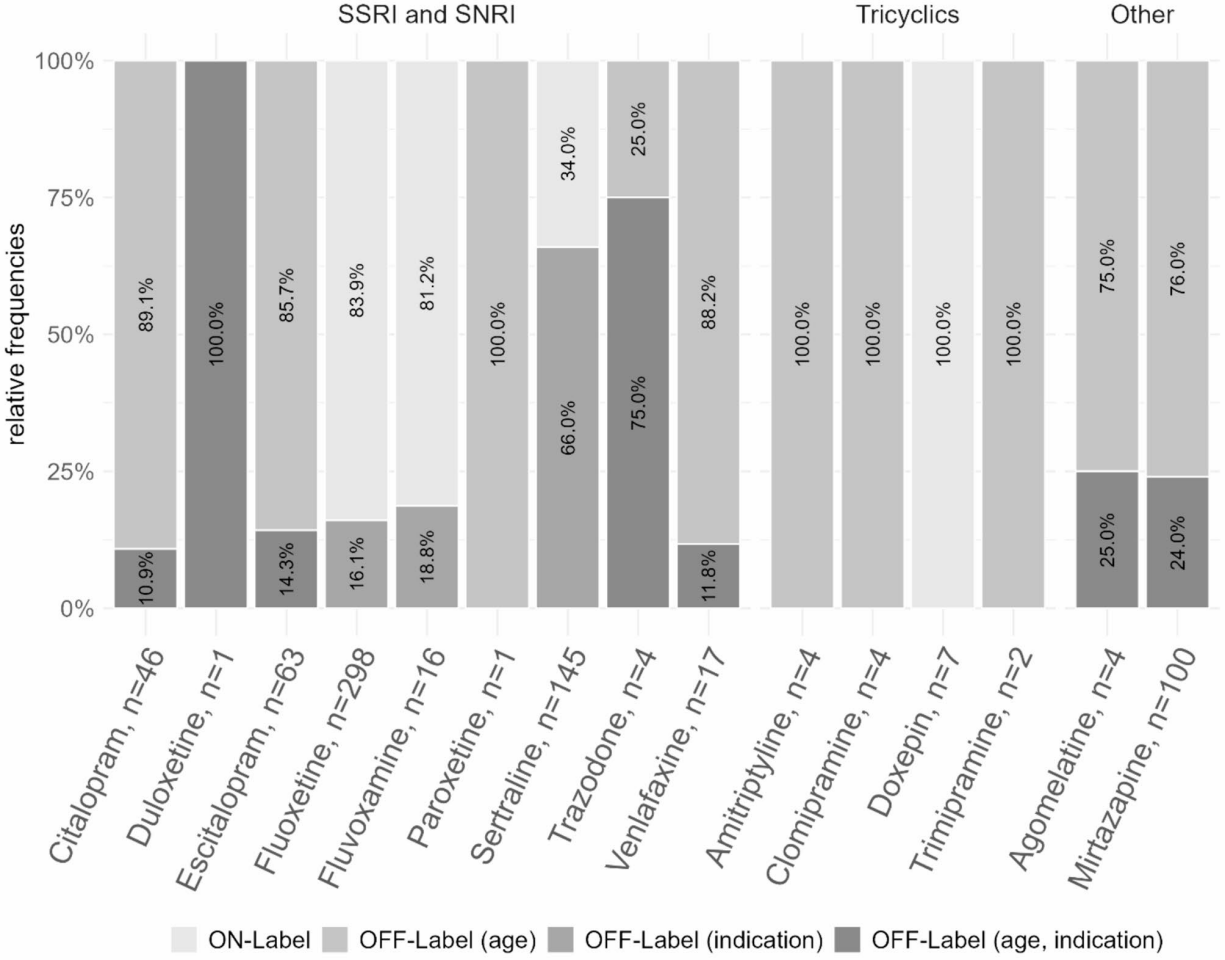
Proportion of treatment episodes with on- vs. off-label antidepressant prescriptions

For the distribution of on- and off-label use for each single antidepressant, see Fig. 2.

### Off-label use of antipsychotics

With a share of 452 (81.7%) of all 553 antipsychotic treatment episodes, off-label use was even greater with antipsychotics than with antidepressants. Prescriptions outside approval occurred roughly equally frequently in subjects ≥ 12 years (81.6%) and in subjects < 12 years old (83.3%). Altogether, 38.3% of the off-label antipsychotic treatment episodes involved patients diagnosed with a depressive episode, 24.1% included patients with eating disorders, and 25.9% were observed in patients with suicidality at baseline. Furthermore, also a trend towards an increased rate of off-label use in patients with hyperkinetic disorders was demonstrated (16.6% of the episodes).

For antipsychotics, off-label use due to age (29.4%, *n* = 133) was more common than off-label use by indication alone (33.2%, *n* = 150), and 37.4% (*n* = 169) of the episodes were off-label due to age and indication.

Of antipsychotic treatment episodes that were off-label due to age, 92.7% were episodes in adolescents, while 7.3% were episodes in children (*n* = 22; mean age 9.7, SD 1.1, median 10.0 years).

### Off-label use of SGAs

For SGAs (*n* = 450 episodes) the proportion of off-label treatment episodes was 92.0% (30.7% by age, 35.5% by indication, 33.8% by age and indication). Aripiprazole was the most commonly prescribed off-label in 80.8% of the treatment episodes, of which 4.1% were in relation to age, 77.9% to indication, and 18.0% due to age and indication.

The next most common SGAs, quetiapine and olanzapine, were administered off-label in 100% of treatment episodes, as they are not licensed in minors in Germany. Risperidone was used outside the approved indication in 100.0% of treatment episodes in patients who were not diagnosed with below average intellectual functioning or mental retardation, and in 77.8% of treatment episodes in patients who were diagnosed with below average intellectual functioning or mental retardation. Altogether, 7.7% (*n* = 4) of the in TDM-VIGIL documented treatment episodes with risperidone were longer than the approved short-term therapy of 6 weeks, in three of these, risperidone was also used outside the approved indication and was therefore assigned to this category.

### Typical antipsychotics

For typical antipsychotics, *n* = 103 treatment episodes were documented. The rate of off-label treatment episodes was 36.9%, mainly related to age and indication (76.3%) and only due to age in 15.8% and due to indication in 7.9% of the episodes.

**Fig. 3:**
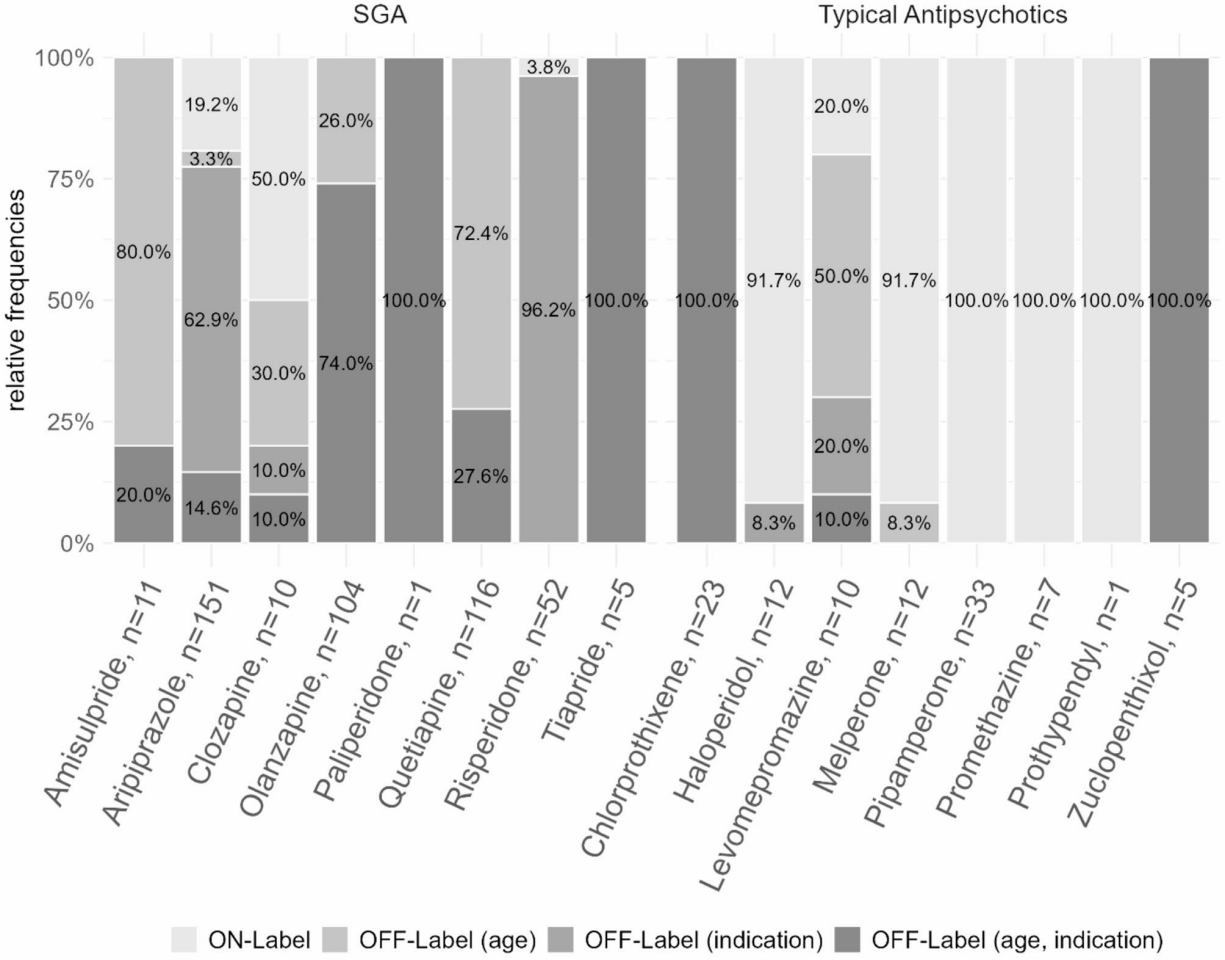
Proportion of treatment episodes with on- vs. off-label antipsychotic prescriptions

For the distribution of on- and off-label use in each single antipsychotic, see Fig. 3.

### Frequency and modulating factors of off-label use

Analyzing possible factors associated with off-label use by descriptive univariate analysis in the whole patient sample, in 70.5% of boys (165 of 234) and in 69.7% of girls (325 of 466) (*p* = 0.903) at least one treatment episode with an antidepressant or antipsychotic was classified as off-label. Altogether, 66.7% (40 of 60) of children (age < 12 years) and 70.3% of adolescents (450 out of 640) (*p* = 0.659) had at least one treatment episode classified as being off-label. Thus, neither sex nor age significantly moderated off-label use. The proportion of patients from university hospitals with at least one off-label episode (72.4% of the patients, 372 of 514) was higher compared to patients from non-university centers (63.4% of, 118 of 186) (*p* = 0.029). Considering intelligence level of the patients, no significant effect on off-label use was observed, as at least one treatment episode was classified as off-label in 69.0% (437 of 633) of patients with average intelligence compared to 77.6% (45 of 58) (*p* = 0.227) of patients with below average intelligence (intelligence status unknown in 9 patients). Similarly, no significant moderating effect was observed for suicidality in the whole patient sample, as 75.1% (133 of 177) of patients with suicidality at baseline and 67.9% (344 of 507) of patients without suicidality (*p* = 0.085) had at least one treatment episode classified as off-label (in 16 patients the status was unknown). In patients with a schizophrenia-spectrum diagnosis at baseline (ICD-10 F2X), 90.2% (55 of 61) had at least one medication episode (antidepressants and/or antipsychotics) classified as off-label, compared to 68.1% (435 of 639) of patients without a schizophrenia spectrum disorder (*p* < 0.001). Finally, in terms of setting, the proportion of patients receiving off-label use pharmacotherapy was 63.6% (35 out of 55) for outpatients, 73.1% (396 out of 542) for inpatients and 57.3% (59 out of 103) for day hospital patients (*p* = 0.003).

In the 10 centers with at least 30 patients (569 patients total), the hospital of Cologne had the lowest proportion of patients with at least one off-label medication episode (18.2%, 6/33), while the University Hospital of Vienna had the highest (98.1%, 51/52). However, the total number of medication episodes confounds the relationship between ‘center’ and the target variable ‘at least one off-label use episode’, resulting in a misleading association between center and off-label use. The ranking of centers by the proportion of patients with off-label use closely matches the ranking by the proportion of patients with more than one medication episode.

Multivariable binary logistic regression analyses were used to explore factors associated with the probability of at least one off-label treatment episode. The variables sex, age, setting, intelligence, suicidality, university hospital status, number of medication episodes and psychiatric diagnoses at baseline were included. Stepwise backward variable selection methods were applied. The number of medication episodes was a significant predictor of at least one off-label use episode. As cross-table analysis revealed this relationship when coding medication episodes as binary (1 versus ≥ 2 ‘medication episodes’), the binary indicator for more than one medication episode was used in the model for clarity. Additionally, the categorized number of medication episodes was significantly associated with age group (< 12 vs. ≥12 years), with number of medication episodes acting as a confounder between age and off-label use.

The Hosmer-Lemeshow test indicated no significant lack of fit for the final model. The AUC under the ROC was 0.874 (95% CI: 0.847–0.901), which corresponds to considerably useful to excellent discriminatory ability between off-label and on-label conditions.

**Table 3:** Regression model: Off-Label-Use in antidepressants plus antipsychotics

Explanatory factor	*p*-value Wald-test	Adjusted Odds Ratio	95% C.I. for OR	
			Lower	Upper
At least one diagnosis ‘depressive episode’	< 0.00001	0.15	0.09	0.25
At least one diagnosis ‘OCD’	< 0.00001	0.09	0.04	0.19
At least one diagnosis ‘eating disorder’	0.002	2.27	1.34	3.84
Suicidality at admission? YES	0.011	1.92	1.16	3.18
More than one medication episode	< 0.00001	29.31	15.15	56.71

OCD = obsessive-compulsive disorder

Table 3 shows that the diagnoses ‘depressive episode’ and ‘OCD’ reduced, the diagnosis/condition of an ‘eating disorder’ and ‘suicidality’ at admission in contrast increased the likelihood of off-label prescription in the whole sample. Additionally, the likelihood of off-label use increased significantly when two or more medication episodes were documented, meaning that adjustments in pharmacotherapy/changes in medication were required.

### Off-label use in antidepressants

Multivariable binary logistic regression analysis was used to explore factors associated with the probability of off-label use of antidepressants in individual episodes. Sex, age, setting, intelligence, suicidality, university hospital status, psychiatric diagnoses at baseline, and the number of the current medication episode in the respective patient were included as predictors. Stepwise backward variable selection methods were applied.

The Hosmer-Lemeshow test indicated no significant lack of fit for the final model. The AUC under the ROC was 0.752 (95% CI: 0.717–0.786), which corresponds to considerably useful to excellent discriminatory ability between off-label and on-label conditions.

**Table 4:** Regression model: Off-Label-Use in antidepressants

Explanatory factor	*p*-value Wald-test	Adjusted Odds Ratio	95% C.I. for OR	
			Lower	Upper
At least one diagnosis ‘depressive episode’	< 0.00001	0.17	0.1	0.3
At least one diagnosis ‘OCD’	< 0.00001	0.06	0.03	0.12
Suicidality at admission? YES	0.035	1.49	1.03	2.17
Number of medication episodes	< 0.00001	1.81	1.46	2.24

OCD = obsessive-compulsive disorder

Table 4 shows that the diagnoses ‘depressive episode’ and ‘OCD’ reduced, the condition of ‘suicidality’ at admission, as well as increasing numbers of medication episodes in contrast increased the likelihood of off-label use of antidepressants.

### Off-label use in antipsychotics

Multivariable binary logistic regression analysis was used to explore factors associated with the probability of off-label use of antipsychotics in individual episodes. Sex, age, setting, intelligence, suicidality, university hospital status, psychiatric diagnoses at baseline, and the number of medication episodes were included as predictors. Stepwise backward variable selection methods were applied.

The Hosmer-Lemeshow test indicated no significant lack of fit for the final model. The AUC under the ROC was 0.698 (95% CI: 0.645–0.751), which corresponds to considerably useful to excellent discriminatory ability between off-label and on-label conditions.

**Table 5:** Regression model: Off-Label-Use in antipsychotics

Explanatory factor	*p*-value Wald-test	Adjusted Odds Ratio	95% C.I. for OR	
			Lower	Upper
At least one diagnosis ‘schizophrenia-spectrum disorder’	< 0.00001	0.27	0.16	0.45
Treatment in a university hospital? YES	0.0026	2.7	1.42	5.15

Table 5 shows that the diagnoses ‘schizophrenia-spectrum disorder’ reduced the likelihood of antipsychotic off-label use, in contrast, treatment in a university hospital increased the likelihood of antipsychotic off-label prescription.

### Symptom severity (CGI-S) and risk for off-label use

Altogether, 465 medication episodes met these criteria for analysis. Multilevel modelling (hierarchical logistic regression) was performed to account for the clustered data structure. Furthermore, the analysis for the effect of CGI-S was adjusted for patient-level fixed factors (baseline characteristics), for which univariate analyses provided some evidence for possible associations with later off-label use with antidepressants or antipsychotics. These patient characteristics were ‘suicidality at admission’, ‘age group’, ‘university or non-university center’, as well as the following psychiatric diagnoses at baseline: ‘depressive disorders’, ‘hyperkinetic disorders’, ‘OCD’ and ‘eating disorders’. Again, the factor ‘specific number of medication episodes’ was included in the modelling (as well ‘1 versus ≥ 2 medication episodes’ as a three level categorization ‘1, 2, ≥ 3 medication episodes’).

The type III test for the fixed factor disease severity showed no significant influence of the severity of illness (categorized CGI-S) on the risk for off-label use (*p* = 0.679 in the case of two level categorization for factor ‘specific number of medication episodes’ and p = 0.696 in the case of three level categorization). However, the specific number of medication episodes, depressive disorders, OCD and suicidality at admission were found to be significant factors influencing the risk of off-label use.

## Discussion

In this prospective naturalistic study, 66.9% of all documented psychotropic treatments (55.2% for antidepressants, 81.7% for antipsychotics) were ‘off-label’, i.e. only about one third of the drug treatment episodes in the studied child and adolescent psychiatric population were administered according to the approved indication, in the approved age group, or according to the licensed treatment duration in case of risperidone.

### Antidepressants

#### Antidepressant prescription patterns

Antidepressants were used to treat a wide variety of indications different from the licensed diagnoses depression (fluoxetine licensed from 8 years on) and OCD (sertraline from 6, fluvoxamine from 8 years on). SSRIs were the predominantly prescribed antidepressants, due to their favorable safety profile, which is consistent with most recent studies on prescription patterns in Germany [24, 25, 64], the European Union (e.g [22, 65]), and worldwide (e.g [13, 66]), and which is also in accordance with national guidelines on the treatment of depression, anxiety disorders and OCD (http://www.awmf.org/leitlinien).

#### Antidepressant off-label use

In our prospective study of mainly inpatients, off-label use of antidepressants was frequent (more than half of all antidepressant treatment episodes), and about 10–15% higher than in the most recent national studies of insurance data of mainly outpatients [25, 64] (dating back to 2004–2011 or 2005–2012), where even a decline of off-label prescriptions from 2004 to 2011 was reported (from 58.0 to 40.9% [24]). Off-label use based on age alone was the reason most common for antidepressant off-label use in TDM-VIGIL (51.1%), a result in line with former national studies [12]. Off-label prescription only by indication was observed in 37.4% of the treatment episodes for the total of antidepressants in TDM-VIGIL, with especially high proportions in specific antidepressants, such as sertraline (66.2%). In contrast to recent studies in Germany [24], the proportion of off-label use in SSRI/SNRI (47.2%) was lower than in TCA (58.8%). The diagnoses ‘depressive episode’ and ‘OCD’ reduced the likelihood of off-label use of antidepressants, the condition of ‘suicidality’ at admission in contrast increased the likelihood of antidepressant off-label prescription.

The interpretation of off-label use in TDM-VIGIL must take into account, that fluoxetine was the most frequently used psychotropic medication, with 83.9% of its use being on-label. Without this, the off-label use of antidepressants would have been significantly higher, as seen in other international studies [13, 67], e.g. studies from Switzerland, were fluoxetine is not licensed in youth and therefore antidepressants showed off-label prescription rates of 100% in 2008 and 2014 [59]. The high amount of on-label fluoxetine prescriptions suggests that marketing authorization influences medication choice, since safety in terms of efficacy and tolerability is documented in the pediatric age group. Reconsidering first-line drugs like fluoxetine may be appropriate only in individual cases due to their specific pharmacological properties, such as fluoxetine’s long half-life.

In the above-mentioned study from Schroder and colleagues [24] the highest antidepressant off-label prescription rates were seen for the use of TCAs in young patients with ADHD. TCAs, however, are not first-line medication for the core symptoms of ADHD and are associated with a high risk of serious cardiac side effects. In TDM-VIGIL, depressive episodes were the most common diagnoses overall, including among patients with off-label episodes. However, about one third of off-label antidepressant treatments referred to patients with eating disorders, and one third to youths with documented acute suicidality at baseline.

### Antipsychotics

#### Antipsychotic prescription patterns

In line with results from recent studies [21, 28, 39–42] the most frequently used antipsychotics in our study were the SGAs aripiprazole, quetiapine and olanzapine. Prescription rates of typical antipsychotics of less than one fifth of all antipsychotic treatments confirmed that child and adolescent psychiatrists adhere to national and international guidelines with a preference of using SGAs in children and adolescents compared to typical antipsychotics [68]. Typical antipsychotics, such as pipamperone, chlorprothixene, melperone and levomepromazine are often prescribed as acute rescue medication due to the marked sedative effects of these substances.

#### Antipsychotic off-label use

Our results showed an immense share of off-label use among antipsychotic treatments (81.9%) with a higher off-label use for SGAs (92.0%) than for typical antipsychotics (36.9%). Antipsychotic off-label prescriptions were rarely only due to indication (33.2%, compared to 37.4% due to age AND indication and 29.4% only due to age) as most SGAs licensed for the treatment of specific child psychiatric disorders in minors are only approved for the treatment of adolescents (aripiprazole 13 years for bipolar disorder, 15 years for schizophrenia; clozapine 16 years for schizophrenia, paliperidone 15 years for schizophrenia). Prescriptions outside approval occurred roughly equally frequently in subjects ≥ 12 years and in subjects < 12 years old, and most often in patients diagnosed with depressive episodes, eating disorders and baseline suicidality. The diagnosis ‘schizophrenia spectrum disorder’ reduced the likelihood of antipsychotic off-label use, whereas treatment in a university hospital increased the risk.

For risperidone, the main reason for off-label prescriptions was the absence of a diagnosis of subaverage intellectual functioning or intellectual disability. Only a small proportion of less than 10% of prescriptions for risperidone for patients with oppositional defiant disorder exceeded the recommended 6-week treatment limit in TDM-Vigil. This for clinicians surprisingly low number could be due to the flexible observation intervals in the study with sometimes very short study follow-up.

In international studies, off-label use of antipsychotics in children reached from about 40% to more than 90% [69] and was associated with diagnoses of attention-deficit/hyperactivity disorder, anxiety, or mood disorders. In the present study, also a trend towards an increased rate of off-label use in patients with hyperkinetic disorder was demonstrated (about 17%). A Danish cross-sectional study from 2014 found similarly high rates (95.5%) of antipsychotic off-label-prescriptions as in TDM-VIGIL, including all inpatients and outpatients at a huge mental health center in the capital region of Denmark, aged 0–17 years (Braüner et al. 2016). In the literature of the last 15 years, risperidone was one of the most frequently off-label used antipsychotics in the pediatric population [69, 70], followed by aripiprazole and quetiapine [9, 69]. In TDM-VIGIL, aripiprazole of all antipsychotics was most commonly used off-label, mainly prescribed outside of approved indications. The shift from a preference towards aripiprazole is likely due to the expected advantages concerning neuromotor and cardiometabolic side effects, and hyperprolactinaemia [71–73].

As already discussed for antidepressants, the additional inclusion of inpatients on antipsychotics likely led to increased off-label use, in our and recent other studies [67], as inpatients probably present with more intensive pharmacological needs.

### Modulating and risk factors of off-label use

In the present study, sex was not a risk factor for off-label use. Age ≥ 12 years was strongly associated with a higher number of medication episodes, confounding the univariate analysis of the association between age group and off-label use. Furthermore, age ≥ 12 years was closely associated with the administration of certain frequently and exclusively off-label used medicines. In detail, nearly all treatment episodes with the exclusively off-label used medicines olanzapine and mirtazapine as well as the majority of episodes with quetiapine and escitalopram treatment occurred in this age group. It might be suggested that prescribers adhere more strictly to the approval for children under 12 years of age due the immense lack of data and fear of possible side effects, while psychotropic drugs tested and approved for adults are more frequently and courageously used for adolescent patients > 12 years old.

Global illness severity, measured with the CGI-S, had no influence on whether the following antidepressant or antipsychotic treatment was off-label. Suicidality at admission increased the likelihood of off-label antidepressant use, likely due to depressive episodes treated with SSRIs other than fluoxetine (such as sertraline, citalopram and escitalopram), which are not approved for this indication. Suicidal crises are also common in borderline personality disorder, for which no approved psychotropic drugs exist.

In TDM-VIGIL, treatment in a university hospital (but not symptom severity) was associated with a higher risk for off-label use of antipsychotics. This result is thus in line with previous reports on a high risk of off-label prescriptions in specialist units compared to e.g. treatment by the general practitioner [12, 30]. Hospital physicians and psychiatrists and even more physicians at university hospitals usually treat amongst others more complex treatment-resistant patients [74], which may contribute to the high rate of off-label use within this setting. Furthermore, the risk of recourse claims related to off-label use may play a significant role in residental care, whereas university hospitals tend to prescribe psychopharmacological drugs off-label more “courageously” based on clinical necessity, as initial treatments are conducted in closely monitored settings, unlike outpatient care.

A diagnosis of an eating disorder increased the risk of off-label treatment episodes in the whole sample, likely due to the lack of approved medications for anorexia’s core symptoms, leading e.g. to off-label prescriptions for symptoms like restless drive for activity, resistance to weight gain, and severe obsessional thinking [29, 75]. Diagnoses of depressive episodes and OCD at baseline reduced the likelihood of off-label treatment episodes in general and when analysing just antidepressant treatment episodes, as SSRIs like fluoxetine, sertraline, and fluvoxamine are approved for these indications in youth. In antipsychotic treatments, the diagnosis ‘schizophrenia spectrum disorder’ reduced the likelihood of off-label use significantly, as there are several approved antipsychotics for this indication in adolescence.

Not surprisingly, multivariable binary logistic regression showed that the number of medication episodes significantly predicted the outcome ‘at least one off-label use episode’. This issue arises due to the medication episode structure of the data in conjunction with the patient-based main outcome ‘at least one episode with off-label drug use’. Any analysis that ignores the ‘number of medication episodes’ (especially univariate analyses, but also multivariable modelling) risks confounding and spurious associations (e.g., fixed effect age).

### Future prospects

While off-label prescribing may be seen as inappropriate, it is important to recognize that the high rate of off-label use of antidepressant and antipsychotics in this pediatric group likely stems from a lack of approved drugs despite efficacy and safety data of that specific medication in adults, or in children and adolescents for other members of the same medication class. In Europe, only a few antidepressants and antipsychotics are approved for pediatric use, leaving clinicians with limited options when approved drugs are ineffective, unsafe, or not covered by insurance. For example, most drugs are developed through adult trials, so prescribing for children and adolescents often relies on extrapolating data (on dosing, side effect profile, etc.) from these studies. However, factors like age, CNS development, comorbidities, comedications, and genetic differences may contribute to varying drug metabolism in pediatric patients. The lack of evidence leads to clinical decisions that are insufficiently backed by evidence. Consequently, pharmaceutical companies must conduct placebo-controlled studies on the efficacy, safety, and dosage of psychotropic drugs in children and adolescents, and funding for head to head trials of antidepressants and antipsychotics is needed.

## Limitations and strengths

Unlike previous studies in Germany that used health insurance data and focused on outpatient settings, TDM-VIGIL provides data from specialized child and adolescent psychiatric sites, mainly in inpatient settings, ensuring more accurate diagnostic and treatment strategies. The data were collected prospectively in a multicenter study using an internet-based registry, allowing for more detailed individual patient information.

Due to specialized treatment in university hospitals and inpatient settings, generalizing the results to all children and adolescents with mental disorders on psychotropic medication is difficult. Additionally, off-label use by dose was not assessed, and treatment episodes from Switzerland were evaluated based on German authorization regulations, despite potential differences in Swissmedic approvals. Lastly, the study was not set up to compare the efficacy and safety of on-label versus off-label prescribed antidepressants and antipsychotics.

## Conclusion

This study demonstrated a high frequency of off-label prescribing of antidepressants (about 55%) and especially of antipsychotics (about 82%) with respect to age and indication in specialized child and adolescent psychiatry centers in three European countries. This frequent off-label prescribing practice highlights the lack of clinical trials and marketing authorization for pediatric use, emphasizing the need for economic and legal incentives for companies to test and register off-patent products for pediatric indications.

## Data Availability

The datasets used and/or analysed during the current study are available from the corresponding author on reasonable request.
